# Early sevoflurane sedation in severe COVID-19-related lung injury patients. A pilot randomized controlled trial

**DOI:** 10.1186/s13613-024-01276-4

**Published:** 2024-03-27

**Authors:** Beatrice Beck-Schimmer, Erik Schadde, Urs Pietsch, Miodrag Filipovic, Seraina Dübendorfer-Dalbert, Patricia Fodor, Tobias Hübner, Reto Schuepbach, Peter Steiger, Sascha David, Bernard D. Krüger, Thomas A. Neff, Martin Schläpfer

**Affiliations:** 1 Institute of Anesthesiology, University Hospital Zurich University of Zurich, Raemistrasse 100, Zurich, CH-8091 Switzerland; 2 Institute of Physiology, University of Zurich, Zurich, Switzerland; 3 Department of Surgery, Rush University, Chicago, IL USA; 4 Division of Anesthesiology, Intensive Care, Rescue and Pain Medicine, Cantonal Hospital St. Gallen, St. Gallen, Switzerland; 5 Institute of Anesthesia and Intensive Care Medicine, City Hospital Triemli, Zurich, Switzerland; 6 Department of Anesthesia and Intensive Care Medicine, Cantonal Hospital Muensterlingen, Muensterlingen, Switzerland; 7 Institute of Intensive Care Medicine, University Hospital Zurich, University of Zurich, Zurich, Switzerland

**Keywords:** Sevoflurane, Volatile anesthetics, Anesthetic conditioning, COVID-19-related lung injury

## Abstract

**Background:**

This study aimed to assess a potential organ protective effect of volatile sedation in a scenario of severe inflammation with an early cytokine storm (in particular IL-6 elevation) in patients suffering from COVID-19-related lung injury with invasive mechanical ventilation and sedation.

**Methods:**

This is a small-scale pilot multicenter randomized controlled trial from four tertiary hospitals in Switzerland, conducted between April 2020 and May 2021. 60 patients requiring mechanical ventilation due to severe COVID-19-related lung injury were included and randomized to 48-hour sedation with sevoflurane vs. continuous intravenous sedation (= control) within 24 h after intubation. The primary composite outcome was determined as mortality or persistent organ dysfunction (POD), defined as the need for mechanical ventilation, vasopressors, or renal replacement therapy at day 28. Secondary outcomes were the length of ICU and hospital stay, adverse events, routine laboratory parameters (creatinine, urea), and plasma inflammatory mediators.

**Results:**

28 patients were randomized to sevoflurane, 32 to the control arm. The intention-to-treat analysis revealed no difference in the primary endpoint with 11 (39%) sevoflurane and 13 (41%) control patients (*p* = 0.916) reaching the primary outcome. Five patients died within 28 days in each group (16% vs. 18%, *p* = 0.817). Of the 28-day survivors, 6 (26%) and 8 (30%) presented with POD (*p* = 0.781). There was a significant difference regarding the need for vasopressors (1 (4%) patient in the sevoflurane arm, 7 (26%) in the control one (*p* = 0.028)). Length of ICU stay, hospital stay, and registered adverse events within 28 days were comparable, except for acute kidney injury (AKI), with 11 (39%) sevoflurane vs. 2 (6%) control patients (*p* = 0.001). The blood levels of IL-6 in the first few days after the onset of the lung injury were less distinctly elevated than expected.

**Conclusions:**

No evident benefits were observed with short sevoflurane sedation on mortality and POD. Unexpectedly low blood levels of IL-6 might indicate a moderate injury with therefore limited improvement options of sevoflurane. Acute renal issues suggest caution in using sevoflurane for sedation in COVID-19.

**Trial registration:**

The trial was registered on ClinicalTrials.gov (NCT04355962) on 2020/04/21.

**Supplementary Information:**

The online version contains supplementary material available at 10.1186/s13613-024-01276-4.

## Background

Numerous studies have investigated the potential of volatile and intravenously applied general anesthetics to protect organs. Clinical trials have highlighted the benefit of using volatile anesthetics during surgery in severe ischemia-reperfusion-induced injury in the heart, the lung, the liver, and the kidneys [1–5]. However, it is still questionable if volatile anesthetics provide organ protection during the sedation of patients in intensive care units (ICU) and if the degree of injury is high enough, as this seems to be a prerequisite for successful protection [6]. A randomized controlled study showed a transient increase in oxygenation in patients with acute respiratory distress syndrome (ARDS) when sedated for 48 h with sevoflurane [7]. The study design of the current trial was based on this previous trial.

The practice of volatile sedation in the ICU is not entirely new. Worldwide, several centers have extensive experience with volatile sedation in the ICU setting [8, 9]. Specific devices available in most high-volume anesthesia departments are required to administer volatile anesthetics in ICUs, such as the Anaesthetic Conserving Device (Sedaconda®) or the MIRUS™ System.

The clinical presentation of patients diagnosed with severe acute respiratory syndrome coronavirus 2 (SARS-CoV-2) COVID-19 infections suffering from a disease entity termed COVID-19 varied in the first epidemic wave. The mortality rate was as high as 80% in mechanically ventilated patients [10]. Organ injury and, finally, the high mortality rate of COVID-19 ARDS were suggested to be related to virally driven hyperinflammation [11].

Hyperinflammatory syndromes in adults with viral infections are well known and often characterized by increased levels of inflammatory mediators such as interleukin-2 (IL-2), IL-6, tumor necrosis factor-α (TNF-α), monocyte chemoattractant protein − 1 (MCP-1) or macrophage inflammatory protein 1-α (MIP-1α) [12, 13]. In an early study performed in COVID patients, IL-6 was significantly increased in non-survivors [11] highlighting the crucial role of this biomarker in inflammation as previously shown [14]. Therefore, during the first wave of the COVID-19 pandemic, we hypothesized that an episode of volatile sedation with sevoflurane might reduce this severe inflammatory response to SARS-CoV-2 by attenuating the cytokine storm. We hypothesized that sevoflurane, with its well-defined anti-inflammatory effect, would improve the 28-day outcome. Because many factors were unknown when the trial was designed, it was set up as a pilot small-scale study to explore the effect of sevoflurane in order to design then a larger trial.

## Methods

The trial was registered on ClinicalTrials.gov (NCT04355962) and is reported according to the consolidated standards of reporting trials (CONSORT) checklist [15]. The trial was approved by the ethics committee and the drug administration authorities; details are indicated in the online supplement.

### Participants

Patients suffering from SARS-CoV-2 infection were screened in four Swiss tertiary hospitals. Inclusion criteria were patients 18 to 85 years old suffering from severe COVID-19-related lung injury (PaO_2_/FiO_2_ < 200mmHg before intubation); sedation and invasive mechanical ventilation on an ICU, intubation < 24 h before study inclusion; electrocardiogram with a corrected QT time < 470ms for male and < 480ms for female patients. Exclusion criteria were corticosteroid intake (equivalent dose of > 10 mg prednisone per day before hospitalization); the presence of significant concomitant disease (e.g., acute cerebrovascular event, acute coronary syndrome, seizure, burn, neuromuscular disease); patient after organ transplantation; acquired immune deficiency syndrome, pregnancy or breastfeeding; the use of a cytokine absorber, suspected or documented lack of consent to the research intervention.

Standardized lung protective ARDS ventilation protocols of the individual centers were followed, but the final ventilation strategy was at the clinician’s discretion.

### Ethics approval and consent to participate

The local ethics committee (Kantonale Ethikkommission Zürich; study ID: 2020 − 00719) and the national authorization and supervisory authority for drugs and medical products (Swissmedic; study ID: 2020DR3050) approved the trial (date: April 9, 2020; study title: “sevoflurane sedation in COVID-19 ARDS patients to reduce lung injury: a randomized controlled trial.)

The local ethics committee (Kantonale Ethikkommission Zürich; study ID: 2020 − 00719) and the national authorization and supervisory authority for drugs and medical products (Swissmedic; study ID: 2020DR3050) approved the trial (date: April 9, 2020; study title: “sevoflurane sedation in COVID-19 ARDS patients to reduce lung injury: a randomized controlled trial”). Because patients were under sedation at the time of enrollment into the trial, the study team obtained consent from an independent physician not involved in this research project which was consulted to protect the patient’s interests. The patient’s legal representative was approached as soon as possible, at least within 7 days after enrollment, and informed about the nature of the trial. Post-hoc written and informed consent was obtained from the patient or the legal representative (in patients not regaining decisional capacity within 7 days). Lack of written consent resulted in study exclusion.

The trial was conducted in accordance with ethical standards, national legislation, and the Helsinki Declaration.

### Interventions

For the interventional arm, patients were assigned to sedation for 48 h with sevoflurane (0.5 to 2.6 vol%), administered by the Sedaconda-L®, the Sedaconda-S® (Sedana Medical, Danderyd, Sweden), or the MIRUS™ system (Medcaptain Medical, Shenzen, China) with the start of the intervention within the first 24 h after intubation. In the control arm, continuous sedation with intravenously infused propofol, midazolam or dexmedetomidine, or a combination of these drugs was performed according to the standard care of the hospitals. Opioids were administered to all patients in both groups. Rescue sedation was achieved by the additional application of midazolam and dexmedetomidine. Due to ethical reasons, applying volatile anesthetics beyond the study intervention was allowed as a last-resort rescue drug. A light sedation was chosen in which the patients tolerated mechanical ventilation.

### Outcomes

The primary endpoint was a composite endpoint of death or persistent organ dysfunction (POD) at day 28. POD was defined as the need for respiratory (invasive mechanical ventilation), cardiovascular (need of vasopressors), or kidney (renal replacement therapy) support. It was based on COVID data available in 2020 [10].

Secondary endpoints were the length of ICU and hospital stay, plasma inflammatory and endothelial biomarkers (C-reactive protein, CRP; procalcitonin (PCT); interleukin-6, IL-6; angiopoietin-1 and − 2; soluble urokinase-type plasminogen activator receptor, suPAR), routine laboratory parameters (creatinine, urea), as well as sex-related differences in adverse events. CRP, PCT, and IL-6 are well-known and characterized markers for severe inflammation. Angiopoietin-1 and − 2, as well as suPAR, are new biomarkers still under investigation, referring to their prediction value for such scenarios [16]. Blood samples for biomarker analyses were taken at study inclusion, thereafter every 24 h until day 8.

The term adverse events in this manuscript reflect all adverse event and serious adverse events reported by the centers according to the official ICH/ WHO definition [17]: “An adverse event is any untoward medical occurrence in a patient or clinical investigation subject administered a medical product and which does not necessarily have a causal relationship with this treatment”. All adverse and serious adverse events were entered as free text. For acute (on chronic) kidney failure the KDIGO [18] or the RIFLE [19] criteria was applied, for liver failure the Clichy criteria [20], for respiratory failure, the necessity of re-intubation.

### Sample size

When designing the trial, very limited data was available on the clinical outcomes of patients with severe COVID-19-related lung injury. Also, so far, no clinical trials limited to viral ARDS have been performed up to now. Therefore, the anti-inflammatory effect of sevoflurane in this context is elusive. Therefore, the sample size calculation was based on assumptions only. The primary endpoint was a composite outcome measure of mortality and POD at day 28.

An early cohort study reported a mortality rate of 81% at 28 days in patients with invasive mechanical ventilation due to severe COVID-19-related lung injury (30 out of 37 patients) [10]. The incidence of respiratory, cardiovascular, and renal dysfunction in these first patients was also high [10, 21]. A proportion of 43% of the survivors (3 out of 7) was still on mechanical ventilation on day 28 [10]. Acute kidney injury (AKI) within the first 28 days after ICU admission was found in 23% of ICU patients [21], renal replacement therapy in 17% [10], and vasopressor support in 35% [10].

The attenuation of hyperinflammation in ARDS by sevoflurane has been described in animal [22–24] and human studies [7]. In contrast, the survival benefit of attenuating hyperinflammation by volatile anesthetics has, up to now, only been demonstrated in septic animals [25, 26]. Based on all these data, we assumed that an intervention-induced reduction of the mortality and POD from 80 to 40% at day 28 would be a realistic intervention effect size. A power analysis with a power of 80%, an alpha-error of 0.05, and a null-difference in proportions of 5% results in a sample size of 29 patients in each arm, or 58 patients in total. Assuming an additional drop-out rate of 10%, a total sample size of 64 patients was calculated. Due to a relatively high drop-out rate during the study it was increased to 20%, ending up with 70 patients (approved amendment by both authorities Cantonal Ethics Committee and Swissmedic).

### Randomization and blinding

Randomization was performed in REDCap using a 1:1 allocation. Stratification for the ventilator type and the study centers was used as different ICU ventilators (turbine and compressed air based) have been used. Patients were blinded as they were not informed about their group assignment.

### Statistical methods

For descriptive statistics, means and standard deviations, median and interquartile range, and percentages were calculated. Normality was tested using the D’Agostino&Pearson test. Differences in the primary outcome, its individual components, and the incidence of adverse events were compared using a ChiSquare test. The duration of ICU and hospital stay were compared using one-way analysis of variance. Between group differences of laboratory parameters were assessed by the Student’s t-test (normally distributed data, two groups), Mann-Whitney test (not normally distributed data, two groups). If a difference was found in the Student’s t-test or the Mann-Whitney test, the authors additionally computed a univariate mixed-effect model to detect whether a significant “time effect” or a “treatment effect” could be detected.

## Results

### Patient flow

**Fig. 1:**
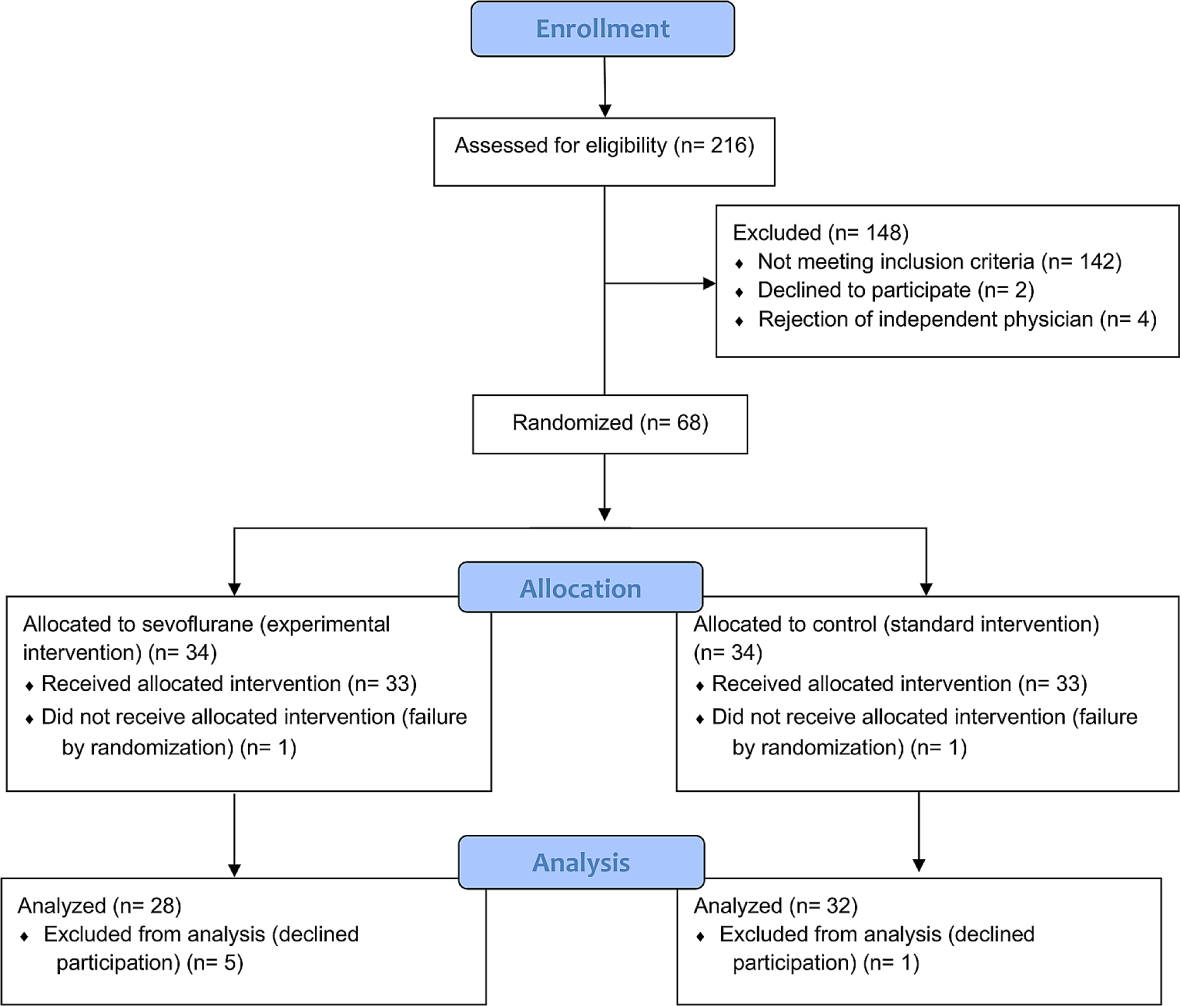
Illustrates the patient flow in this study

Two hundred sixteen patients were screened for eligibility between April 21, 2020, and May 26, 2021. Sixty-eight patients were randomized. The intention-to-treat analysis was performed on 28 patients in the intervention group and 32 in the control group. Details about the patient flow are indicated in Fig. 1.

The last patient completed the trial on June 25, 2021. Information about the primary endpoint on day 28 was complete for all patients.

### Baseline characteristics

The study population comprised 8 (29%) females in the sevoflurane and 10 (31%) in the control group. Age, BMI, and comorbidities were comparable in the two groups as indicated in Table 1. Of note, there was a higher percentage of patients taking ACE inhibitors at baseline in the sevoflurane group, which is relevant given the theorized presumed involvement of the ACE receptor in the pathogenesis of COVID 19. The PaO_2_/FiO_2_ index before sevoflurane or control treatment was 147±53 vs. 167±60 mmHg. Details are depicted in the online supplement, Fig S1.

**Table 1:** Patient characteristics

Variable	Sevoflurane	Control
*Patient characteristics*	*n* = 28	*n* = 32
Age (y), mean (± SD)	61 ± 11	63 ± 11
Sex - male, n (%)	20(71)	22(69)
BMI (kg/m^2^), mean (± SD)	31 ± 6	31 ± 6
Comorbidities, n (%)	20(71)	26(81)
Hypertension, n (%)	11 (39)	18(56)
Other cardiac (Coronary heart disease, arrhythmias), n (%)	3 (11)	4 (13)
Diabetes, n (%)	6 (21)	7 (22)
Chronic kidney disease, n (%)	1 (4)	3 (9)
Active cancer, n (%)	2 (7)	0(0)
Others, n (%)	12 (43)	14(44)
*Concomitant medication (at study inclusion)*		
NSAID, n (%)	2 (7)	0(0)
Antidiabetic oral, n (%)	3 (11)	4 (13)
ACE inhibitor, n (%)	18(64)	10 (31)
Dexamethasone, n (%)	25(89)	31(97)
*Analgosedation (at study inclusion)*		
Propofol, n (%)	27(96)	27(84)
Clonidine, n (%)	1 (4)	0(0)
Dexmedetomidine, n (%)	3 (11)	5 (16)
Midazolam, n (%)	7 (25)	9 (28)
Sevoflurane, n (%)	0(0)	0(0)
Isoflurane, n (%)	0(0)	0(0)
Ketamine, n (%)	1 (4)	0(0)
Fentanyl, n (%)	28(100)	30(94)
Sufentanil, n (%)	0(0)	1 (3)
Morphine, n (%)	1 (4)	0(0)
*Ventilation settings at baseline*		
PEEP (cmH_2_O), mean (± SD)	12 ± 2	12 ± 5
MV (l/min), mean (± SD)	10.9 ± 2.8	9.7 ± 3.0
PaO_2_/FiO_2_ (mmHg), mean (± SD)	147 ± 53	167 ± 60

Table 1 Data are presented as mean ± standard deviation (SD) or as absolute numbers (n) and percentage (%). Abbreviations: y: years; kg: kilogram; m^2^: square meters; BMI: body mass index; NSAID: non-steroidal anti-inflammatory drugs; ACE: angiotensin-converting enzyme, dexamethasone; PEEP: positive end-expiratory pressure; MV: minute ventilation; PaO_2_: arterial partial oxygen pressure; FiO_2_: fraction of inspired oxygen. All parameters are reported at the time of study inclusion

### Study intervention and post-intervention phase

During the 48 h-study intervention, all but one patient received sevoflurane in the sevoflurane arm. The mean sevoflurane dose was 1.2±0.4 etVol%. Additionally, 4 patients received propofol, 4 dexmedetomidine, 12 midazolam, 1 ketamine, and all patients fentanyl. In the intravenous group, 28 patients received propofol, 12 dexmedetomidine, 2 clonidine, 12 midazolam, 2 ketamine, 30 fentanyl, and 1 sufentanil. None of the patients in the intravenous group received sevoflurane during the intervention phase.

Sedation was titrated, so that patients tolerated mechanical ventilation. During the study intervention, the average Richmond agitation sedation score was − 4.4 in the sevoflurane vs. -3.9 in the propofol group.

After the intervention phase, sevoflurane was continued in 15 patients in the sevoflurane group (mean total exposure in these 15 patients: 104 h) and was installed in 6 patients in the intravenous group (mean total exposure: 57 h).

### Primary outcome

The primary endpoint was reached by 11 patients (39%) in the sevoflurane and by 13 (41%) in the control group (*p* = 0.916) (Table 2). The death rate was similar with 5 patients (16%) in the intervention group and 5 (18%) in control (*p* = 0.817).

**Table 2:** Primary and secondary outcomes

Variable	Sevoflurane	Control	p-value
*Primary outcome*	*n* = 28	*n* = 32	
Primary endpoint reached, n (%)	11 (16)	13 (41)	0.916
Death, n (%)	5 (16)	5 (18)	0.817
* Survivors*	*n* = 23	*n* = 27	
Persistent organ dysfunction (POD), n (%)	6 (26)	8 (30)	0.781
Need of mechanical ventilation	6 (26)	8 (30)	0.781
Need of vasopressors	1 (4)	7 (26)	**0.028**
Need of renal replacement therapy	2 (9)	4 (15)	0.502
*Secondary outcomes*	*n* = 28	*n* = 32	
Length of stay in ICU, days	17(± 8)	17(± 9)	0.607
Length of stay in hospital, days	23(± 6)	21(± 7)	0.808
Overall adverse events	23(82)	20(63)	0.088
Respiratory failure	3 (11)	2 (6)	0.533
Cardiac arrythmia	7 (25)	8 (25)	1.000
Congestive heart failure	1 (4)	0	0.214
Acute kidney injury	11 (39)	2 (6)	**0.001**
Acute liver injury	1 (6)	3 (9)	0.356
Sepsis	7 (25)	4 (13)	0.211
Nosocomial infection	11 (39)	12 (38)	0.887
Delirium	12 (43)	12 (38)	0.673
Adynamic Ileus	5 (18)	1 (3)	0.050
Rhabdomyolysis	5 (18)	4 (13)	0.562

Bold characters represent sigificant p-values, i.e., p < 0.05. Table 2 Data are presented as absolute numbers (n) and percentage (%). Abbreviations: POD: persistent organ dysfunction; ICU: intensive care unit.Adverse events were recorded on day 28, referring to the statements in the patient’s medical record

### Secondary outcomes

#### Individual components of the primary outcome

Of the survivors at day 28 (*n* = 23 sevoflurane, *n* = 27 control), 6 patients (26%) in the sevoflurane arm and 8 (30%) in the control arm presented with POD (*p* = 0.781) (Table 2). Six (26%) and 8 (30%) patients (sevoflurane vs. control) needed mechanical ventilation (*p* = 0.781). One (4%) and 7 (26%) study subjects (*p* = 0.028) were supported by vasopressors in the sevoflurane and control group, respectively, with a 6.5 times higher need for vasopressor support in the control group. Two (9%) and 4 (15%) patients were under renal replacement therapy (*p* = 0.502). While the need for vasopressor support was 6.5 times higher in the control group, there was no difference regarding the other primary outcome parameters. Norepinephrine administration during the first 8 days is illustrated in the online supplement, Fig S3.

#### ICU and hospital stay

Length of stay in the ICU and the hospital was comparable with 17±8 vs. 17±9 and 23±6 vs. 21±7 days in the sevoflurane and control group (*p* = 0.607 and *p* = 0.808, respectively) (Table 2).

#### Adverse events

Overall, 23 (82%) vs. 20 (63%) patients (sevoflurane vs. control) experienced adverse events (*p* = 0.088). The complication rate for cardiac, respiratory, neurological, and other events was not different in the two arms except for AKI. All adverse events and serious adverse events were documented and reported to Swissmedic, the Swiss Agency for Therapeutic Products.

#### Complication AKI

Within the entire study duration of up to 28 days, AKI was diagnosed using the RIFLE or KDIGO criteria. Eleven patients (39%) in the sevoflurane and 2 (6%) in the control group experienced AKI (*p* = 0.001) (Table 2), while the necessity of renal replacement therapy at day 28 was similar (*p* = 0.502). To better understand the high rate of AKI in the sevoflurane group, we analyzed how many patients received prolonged sevoflurane sedation by day 8. Of the 11 patients with AKI in the intervention group, 5 patients (45%) were exposed to sevoflurane for more than 48 h (144, 168, 96, 120, and 120 h). The patients with AKI from the control group did not receive sevoflurane.

**Fig. 2:**
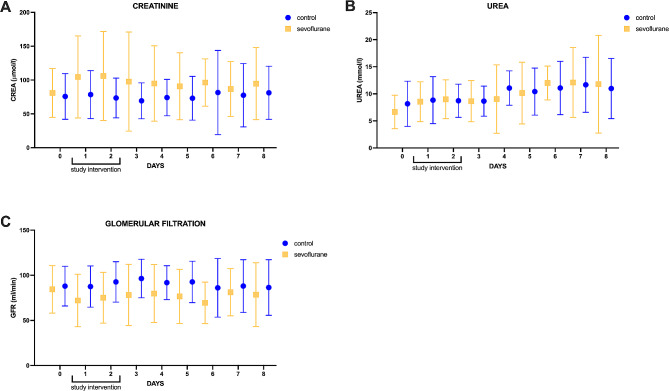
Highlights renal plasma markers up to day 8 in patients subjected to continuous intravenous sedation or a 48 h intervention with sevoflurane. Plasmatic creatinine concentration (2 **A**), urea concentration (2 **B**), and glomerular filtration rate (2 **C**) are illustrated. Values from patients undergoing renal replacement therapy have been excluded. The dots represent mean values; error bars indicate the standard deviation

Creatinine levels (mean±SD, up to day 8) were higher in the sevoflurane group (*p* < 0.001) with comparable starting values (sevoflurane 81±36 vs. control 76±34µmol/l). Values leveled off on day 8 (Fig. 2A). Urea values were similar in both arms (*p* = 0.808) (Fig. 2B). There was a significant difference between the calculated GFR with lower values in the sevoflurane group (*p* < 0.001) with an alignment of the values on day 8 (Fig. 2C). A mixed effects analysis revealed a treatment effect for creatinine only (*p* = 0.024). All patients with preexisting renal failure (3 in control and 1 in sevoflurane) died within 28 days.

#### Adverse events by sex

14 out of 18 (78%) female patients vs. 29 out of 42 (69%) male patients (*p* = 0.485) experienced at least one complication. Of the 8 females in the sevoflurane group, 7 (88%) had an adverse event vs. 7 out of 10 (70%) females in the control group. The incidence of adverse events in male patients was 16 out of 20 (80%) in the sevoflurane group and 13 out of 22 (59%) in the control group.

#### Inflammatory mediators

The following mediators were determined: CRP, PCT, IL-6, angiopoietin-1, angiopoietin-2, and suPAR. Co-medication of drugs potentially impacting the inflammatory reaction are presented in the online supplement, Table S1.

CRP over time was higher in the sevoflurane group (*p* = 0.018). No group difference was observed for PCT (*p* = 0.519) (online supplement, Fig S2A and S2B).

**Fig. 3:**
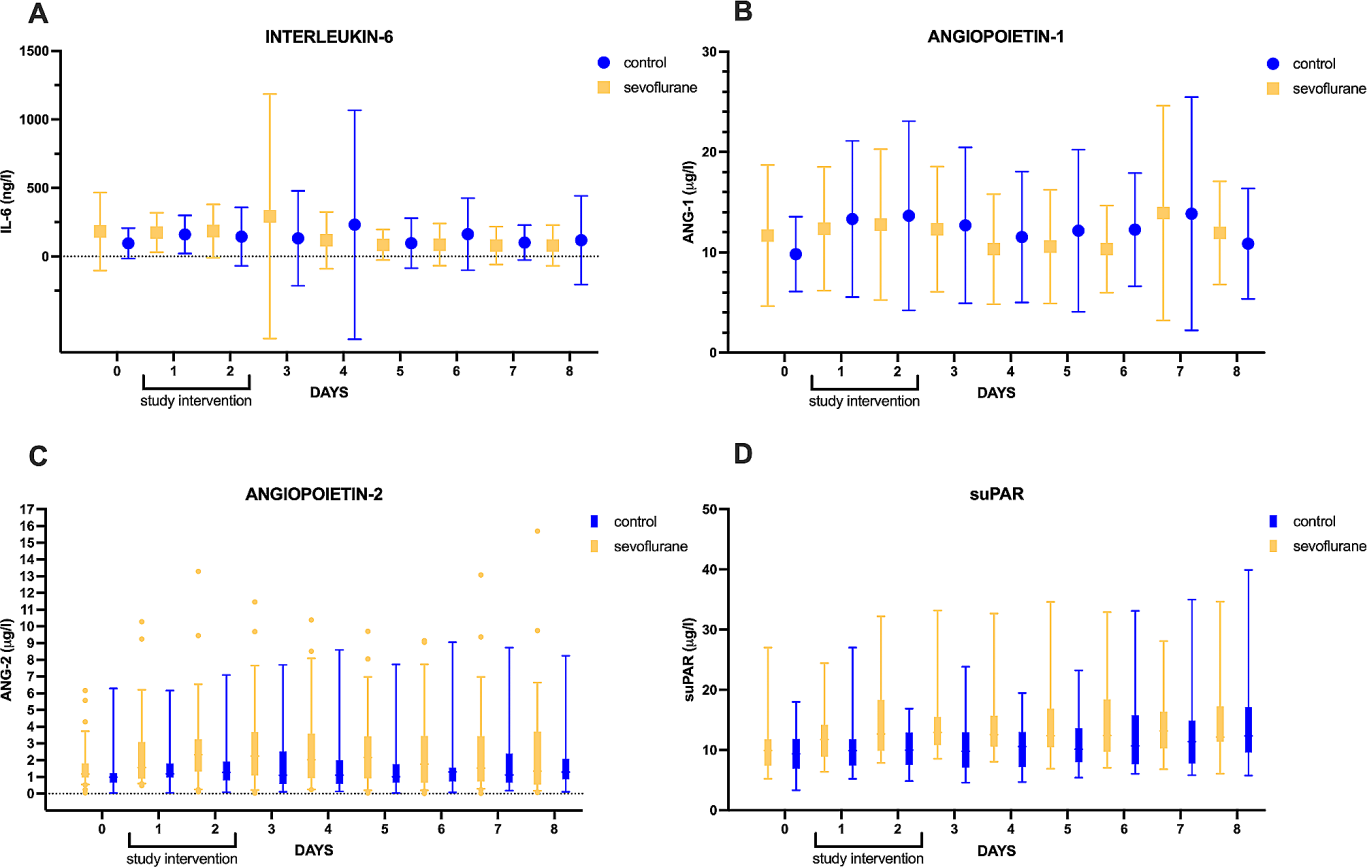
Shows the daily plasma values of inflammatory markers up to day 8 in patients subjected to continuous intravenous sedation or a 48 h intervention with sevoflurane. Interleukin 6 (IL-6, 3 **A**), angiopoietin-1 and − 2 (Ang-1 and Ang-2, 3 **B** and 3**C**), and soluble urokinase-type plasminogen activator receptor (suPAR, 3**D**) are illustrated. The dots represent mean values, error bars indicate the standard deviation (3**A** and 3**B**). Boxplots represent median and 25–75 percentiles, whiskers indicate 10–90 percentiles (3**C** and 3**D**)

Interleukin-6 values did not differ between the two groups (*p* = 0.886) (Fig. 3A) and were found between 79±149 and 293±892 ng/l in the sevoflurane vs. 96±111 and 232±836 ng/l in the control group (mean±SD). Similarly, no difference in angiopoietin-1 expression was detected (*p* = 0.475) (Fig. 3B). Of note are the relatively pronounced standard deviations in both groups (mean±SD): 10.3±5.5 vs. 9.8±3.7 µg/l, max: 13.9±10.7 vs. 13.8±11.6 µg/l in the sevoflurane vs. the control group). In the sevoflurane group, angiopoietin-2 was significantly higher over time (*p* = 0.002) (Fig. 3C), while baseline values (median + IQR) were comparable (sevoflurane: 1.2 (1.0 to 1.8) µg/l, control: 1.0 (0.6 to 1.2) µg/l).

suPAR values (median + IQR) presented similarly at baseline with 9.9 (7.4 to 11.8) µg/l (sevoflurane) and 9.4 (6.9 to 11.9) µg/l (control) (Fig. 3D), respectively, and were again higher after intervention with sevoflurane group (*p* = 0.033) than compared to control.

A mixed effects model revealed a treatment effect for suPAR only (*p* = 0.033).

#### Per protocol analysis

Result of the *per protocol analysis* were comparable to the intention to treat analysis (online supplement Table S2).

#### Additional results

In the online supplement Table S3 further data of the study are found.

## Discussion

This is the first pilot randomized clinical trial to prospectively evaluate the effect of sevoflurane in COVID-19-related lung injury patients requiring invasive mechanical ventilation. A sample size calculation was performed based on the early COVID-19 data available, but in retrospect, the study was underpowered. Therefore, the significant findings should be interpreted with caution. All patients enrolled in the study were not vaccinated, as the COVID-19 vaccination was approved shortly before the end of the study recruitment.

The main findings referring to the predefined outcome parameters can be summarized as follows: A similar composite primary outcome was found for both groups. The need for vasopressor support was lower in the sevoflurane group on day 28. AKI as a complication was more frequently detected in the sevoflurane group, which is in line with the higher creatinine levels during and shortly after the intervention. Finally, suPAR, angiopoietin-2, and CRP plasma levels were higher in the sevoflurane group.

Based on clinical trials with pronounced ischemia-reperfusion injuries in cardiac [27], liver [1, 2], or lung surgery [3, 28], in which the inflammatory response was attenuated by volatile anesthetics, we hypothesized that sevoflurane application would protect organs from severe injury, as these agents seemed to improve clinical outcomes [1, 2, 29], however not in all trials [30, 31].

A main finding of this study, besides the primary and secondary outcome parameters, is the lower-than-expected inflammation. Our trial was designed based on the reported cytokine storm in COVID-19 patients [12], which in the meantime is supported by further literature [32, 33].

These moderately increased levels of inflammatory mediators, in particular the lack of pronounced early peak IL-6 plasma levels, are an important finding of our study, which, of course, was not anticipated by the authors. Also, a recent prospective observational study highlighted lower IL-6 and IL-8 levels in the early phase (days 1–4) of COVID-19-ARDS compared to non-COVID-19-ARDS [34]. Moreover, recent literature does not support the linkage of cytokine storm to COVID-19 ARDS [35]. The less-than-expected inflammation, especially the lack of an early IL-6 peak after ARDS onset, could explain the missing anti-inflammatory and overall organ-protective effect of sevoflurane in this study. Another explanation could refer to a different protective mechanism in bacteria compared to virus-induced ARDS. Typically, pneumonia treated in a hospital is more often caused by bacteria than by viruses. A previous study by Jabaudon et al. has suggested advantageous effects of sevoflurane in this ARDS [7] along with an attenuated level of the soluble receptor for advanced glycation endproducts (sRAGE). Most patients in Jabaudon’s study suffered from ARDS based on pneumonia [7], which we assume was caused by bacteria. However, our study presents a contrasting perspective; although we did not measure sRAGE, the inflammatory markers evaluated in our trial did not suggest a reduction in inflammation. In fact, they indicated a potential exacerbation of the inflammatory response.

An interesting observation refers to the suPAR levels. suPAR is dramatically elevated in patients suffering from severe COVID-19 [36]. Elevated suPAR is associated with COVID-19-related respiratory failure [37] and may even predict AKI in COVID-19 patients [38]. In the current trial the higher suPAR plasma levels and the higher incidence of AKI in sevoflurane-exposed patients align with the Azam data [38]. It is unclear if suPAR is a biomarker or a causative factor [39, 40].

As with all studies, the work presented here has strengths and weaknesses. An evident strength of the trial is that patients were recruited during the first and the second wave of the COVID-19 pandemic in Switzerland; all included patients were unvaccinated. This potentially crucial confounder is therefore absent. Moreover, besides a clinical 8-day follow-up also, biochemical and inflammatory markers were determined, providing a complete picture of the interventional effect, but also the course of the disease was assessed. A limitation is the sample size calculation based on early reported data. Those data indicate a mortality rate of 81% [10]. In the course of the pandemic, a global literature survey finds a mortality rate of 59% for ventilated COVID-19 ARDS patients [41]. More recent data highlight a mortality rate of 25–30% in patients admitted to the ICU for COVID-19 ARDS, which is much closer to the mortality rate observed in the current trial [42, 43].

Based on data from this pilot and the available literature about COVID-19 ARDS the authors do not see an indication to design a larger follow-up study with a sevoflurane intervention in this patient population. Other study scenarios using volatile anesthetics for lung or cardiac protection in the perioperative phase [30, 31] failed to show a beneficial effect of these anesthetics, probably because a severe injury-induced inflammation was missing. Our study results contrast those of previous studies in which sevoflurane was used for organ protection. The results of CRP, suPAR, and the increased incidence of AKI could even be interpreted as an indication of the damaging influence of sevoflurane in COVID-19-related acute lung injury, even if the study has not been powered for these endpoints. However, severe sepsis could be a possible target for a new trial design.

## Conclusion

In conclusion, data from this randomized, controlled pilot study show that a 48-hour sevoflurane intervention does not improve the clinical outcome on day 28 in patients with severe COVID-19-related lung injury. At the same time, the study provides evidence that a pronounced and expected early IL-6 elevation was absent in these patients. This moderate inflammation could be a possible explanation for the lack of protective effect of the sevoflurane intervention. The secondary endpoints (CRP, Ang-2, and suPAR elevation and a higher incidence of AKI) might even suggest an undesired impact of sevoflurane in patients with COVID-19-related lung injury.

### Electronic supplementary material

Below is the link to the electronic supplementary material.

Supplementary Material 1

## Data Availability

Anonymized datasets are available from the corresponding author upon reasonable request.

## Abbreviations

ACE: Angiotensin converting enzyme
AKI: acute kidney injury
ANOVA: analysis of variance
ARDS: acute respiratory distress syndrome
BMI: body mass index
CI: confidence interval
CONSORT: consolidated standards of reporting trials
COVID-19: coronavirus disease 2019
CRP: C-reactive protein
FiO_2_: fraction of inspired oxygen
ICU: intensive care unit
IL: interleukin
IQR: interquartile range
MCP-1: monocyte chemoattractant protein 1
MIP-1α: macrophage inflammatory protein 1 alpha
NSAID: non-steroidal anti-inflammatory drug
PaO_2_: partial pressure of arterial oxygen
PCT: procalcitonin
POD: persistent organ dysfunction
SARS-CoV-2: severe acute respiratory syndrome coronavirus type 2
SD: standard deviation
suPAR: soluble urokinase plasminogen activator receptor
TNF-α: tumor necrosis factor alpha
