# Correction: Early sevoflurane sedation in severe COVID19-related lung injury patients. A pilot randomized controlled trial

**DOI:** 10.1186/s13613-024-01322-1

**Published:** 2024-06-16

**Authors:** Beatrice Beck-Schimmer, Erik Schadde, Urs Pietsch, Miodrag Filipovic, Seraina Dübendorfer-Dalbert, Patricia Fodor, Tobias Hübner, Reto Schuepbach, Peter Steiger, Sascha David, Bernard D. Krüger, Thomas A. Neff, Martin Schläpfer

**Affiliations:** 1 Institute of Anesthesiology, University Hospital Zurich University of Zurich, Raemistrasse 100, 8091 Zurich, Switzerland; 2 Institute of Physiology, University of Zurich, Zurich, Switzerland; 3 Department of Surgery, Rush University, Chicago, IL USA; 4 Division of Anesthesiology, Intensive Care, Rescue and Pain Medicine, Cantonal Hospital St. Gallen, St. Gallen, Switzerland; 5 Institute of Anesthesia and Intensive Care Medicine, City Hospital Triemli, Zurich, Switzerland; 6 Department of Anesthesia and Intensive Care Medicine, Cantonal Hospital Muensterlingen, Muensterlingen, Switzerland; 7 Institute of Intensive Care Medicine, University Hospital Zurich, University of Zurich, Zurich, Switzerland

**Correction: Annals of Intensive Care (2024) 14:41** 10.1186/s13613-024-01276-4

In Table 2 of this article [1], the data in the [Primary endpoint reached, n (%)] headed **Sevoflurane**, should be listed as 11 (39) not 11 (16).

Primary Endpoint Reached, it reads, that 11 patients in the sevoflurane and 13 in the propofol group reached the primary endpoint. This is correct, but the percentages are incorrect, they should be 39 and 41%, respectively.

Table 2 Primary and secondary outcomes (uncorrected)

**Table:** 

Variable	Sevoflurane	Control	p-value
*Primary outcome*	*n* = 28	*n* = 32	
Primary endpoint reached, n (%)	11 (16)	13 (41)	0.916
Death, n (%)	5 (16)	5 (18)	0.817
*Survivors*	*n*** = ***23*	*n*** = ***27*	

2: Primary and secondary outcomes (corrected)

**Table:** 

Variable	Sevoflurane	Control	p-value
*Primary outcome*	*n* = 28	*n* = 32	
Primary endpoint reached, n (%)	11 (39)	13 (41)	0.916
Death, n (%)	5 (16)	5 (18)	0.817
*Survivors*	*n*** = ***23*	*n*** = ***27*	

The original article has been corrected.
