# Supplementary material for: Early sevoflurane sedation in severe COVID-19-related lung injury patients. A pilot randomized controlled trial
Source: Ann Intensive Care. 2024 Mar 27;14:41. doi: 10.1186/s13613-024-01276-4 (PMC10973324; doi:10.1186/s13613-024-01276-4)
Supplement: Supplementary file 1 — Supplementary Material 1 [file 13613_2024_1276_MOESM1_ESM.docx]

# Early sevoflurane sedation in severe COVID-19-related lung injury patients. A pilot randomized controlled trial.

**Online Supplement**

**Authors:**

Beatrice Beck-Schimmer, MD ^1, 2^, Erik Schadde, MD ^2, 3^, Urs Pietsch, MD ^4^, Miodrag Filipovic, MD ^4^, Seraina Dübendorfer-Dalbert, MD ^5^, Patricia Fodor, MD ^5^, Tobias Hübner, MD ^6^, Reto Schuepbach, MD ^7^, Peter Steiger, MD ^7^, Sascha David, MD ^7^, Bernard D. Krüger, MD ^1^, Thomas A. Neff, MD ^6^, Martin Schläpfer, MD ^1,2^

**Affiliations**:

| ^1^ | Institute of Anesthesiology, University Hospital Zurich, University of Zurich, Zurich, Switzerland |  |
| --- | --- | --- |
| ^2^ | Institute of Physiology, University of Zurich, Zurich, Switzerland |  |
| ^3^ | Department of Surgery, Rush University, Chicago, Illinois, USA |  |
| ^4^ | Division of Anesthesiology, Intensive Care, Rescue and Pain Medicine, Cantonal Hospital St. Gallen, St. Gallen, Switzerland |  |
| ^5^  ^6^ | Institute of Anesthesia and Intensive Care Medicine, City Hospital Triemli, Zurich, Switzerland  Department of Anesthesia and Intensive Care Medicine, Cantonal Hospital Muensterlingen, Muensterlingen, Switzerland |  |
| ^7^ | Institute of Intensive Care Medicine, University Hospital Zurich, University of Zurich, Zurich, Switzerland |  |

**Corresponding author**:

Martin Schläpfer, MD, M.Sc., EDAIC

Institute of Anesthesiology

University Hospital Zurich

University of Zurich

Raemistrasse 100

CH-8091 Zurich

Switzerland

Mail: martin.schlaepfer@uzh.ch

| **Content** | **Page** |
| --- | --- |
| Material and methods : Trial design; Determination of inflammatory markers | 3 |
| Results of the per protocol analysis | 5 |
| Results: Sedation details and rescue sedation | 6 |
| Supplementary Table S1 | 7 |
| Supplementary Table S2  Supplementary Table S3  Supplementary Figure S1 | 8  9  10 |
| Supplementary Figure S2 | 11 |
| Supplementary Figure S3 | 12 |
| Study protocol (submitted to the ethics committee), V1.8 | 13 |

**Material and Methods**

**Trial design: authorities and registration**

The local ethics committee (Kantonale Ethikkommission Zürich; study ID: 2020-00719) and the national authorization and supervisory authority for drugs and medical products (Swissmedic; study ID: 2020DR3050) approved the trial (date: April 9, 2020; study title: “sevoflurane sedation in COVID-19 ARDS patients to reduce lung injury: a randomized controlled trial”). Because patients were under sedation at the time of enrollment into the trial, the study team obtained consent from an independent physician not involved in this research project which was consulted to protect the patient's interests. The patient's legal representative was approached as soon as possible, at least within 7 days after enrollment, and informed about the nature of the trial. Post-hoc written and informed consent was obtained from the patient or the legal representative (in patients not regaining decisional capacity within 7 days). Lack of written consent resulted in study exclusion.

The trial was registered on ClinicalTrials.gov (NCT04355962) and is reported according to the consolidated standards of reporting trials (CONSORT) checklist (1). The trial was conducted in accordance with ethical standards, national legislation, and the Helsinki Declaration.

**Trial design**

The last patient completed the trial on June 25, 2021. Information about the primary endpoint on day 28 was complete for all patients. All patients screened and enrolled in the study were not vaccinated, as the COVID-19 vaccination was approved shortly before the end of the study recruitment.

**Determination of inflammatory markers**

C-reactive protein and procalcitonin values were determined as part of the routine analyses in the hospital laboratories of the respective centers. Plasma samples obtained up to day 8 were aliquoted in the laboratories of the respective centers and stored at -80°C for further analysis. Interleukin-6 (IL-6) values have been determined at the Institute of Clinical Chemistry, University Hospital Zurich, Zurich, Switzerland, using the Elecsys IL-6 Kit on a Cobas Analyzer (both from Roche Diagnostics, Rotkreuz, Switzerland). Angiopoietin-1, angiopoietin-2, and the concentration of the soluble urokinase-type plasminogen activator receptor (suPAR) were measured using an enzyme-linked immune sorbent assay (ELISA) from R&D systems (Bio-Techne corporation, Minneapolis, Minnesota, US; catalog numbers DY923, DY623, and DY807) according to the manufacturer’s instructions.

**Reference**

1. Schulz KF, Altman DG, Moher D, Group C. CONSORT 2010 Statement: updated guidelines for reporting parallel group randomised trials. BMC Med. 2010;8:18.

**Results**

**Results of the per-protocol analysis**

As some patients were difficult to become sufficiently sedated, physicians were allowed to use sevoflurane according to their standard procedure. This on top sedation by sevoflurane was applied in 6 patients of the control group (19%) and in 15 of the interventional group during the follow-up phase (54%), in the latter leading to an overall extended sevoflurane exposure time. A per-protocol analysis was performed, including 28 patients from the sevoflurane and 26 from the control group (n=54 in total). The following results were revealed: In the sevoflurane arm, 11 patients (39%) reached the primary endpoint at day 28, and 8 (31%) in the control arm (p=0.512) (**online supplement, Table S2**). The death rate in the intervention group was 18% (5 patients), whereas 15% with 4 patients in the control group (p=0.807).

On day 28, 23 survivors were found in the sevoflurane and 22 patients in the control group. Of these patients, 6 (26%) in the sevoflurane arm and 4 (18%) patients in the control arm presented with POD (p=0.522) (**online supplement, Table S2**). Mechanical ventilation was found in 6 (26%) and 4 (18%) patients in the sevoflurane and control group, respectively (p=0.522), which is in line with the intention-to-treat analysis with all patients reaching the endpoint POD being under mechanical ventilation. Vasopressors were found in 1 (4%) and 4 (18%) patients in the sevoflurane and control group (p=0.129), respectively, and 2 (9%) and 1 (5%) (p=0.573) were under renal replacement therapy.

**Results: Sedation details and rescue sedation**

All patients in the control arm received propofol (2.4±0.3 mg/kg/h). The mean fentanyl dose was 50±4 *vs.* 55±5 μg/kg/h in the sevoflurane *vs.* the control group. During the study intervention (first 48h) additional rescue-sedation was required in both arms. In the sevoflurane arm, 5 patients received additional propofol, 4 patients dexmedetomidine, and 11 patients midazolam. In the control arm, 9 patients received dexmedetomidine, 2 clonidine, and 10 midazolam.

**Table S1 Concomitant medication during the first 8 days of the study**

|  | **Sevoflurane (n=28)** | **Control (n=32)** |
| --- | --- | --- |
| Steroids, n (%) | 28 (100) | 32 (100) |
| Antibiotics, n (%) | 25 (89) | 30 (94) |
| Anti-virals, n (%) | 6 (21) | 8 (25) |
| Anti-mycotics, n (%) | 3 (11) | 0 (0) |
| IL-6 receptor antagonist, n (%) | 0 (0) | 1 (3) |

**Table S1** presents concomitant medication (day 1-8) with a potential impact on the course of inflammation. The steroids applied were methylprednisolone and prednisone. The antibiotics were aminopenicillins, beta-lactam inhibitors, carbapenems, cephalosporins (2^nd^ and 3^rd^ generation), diaminopyrimidines, fluoroquinolones, glycopeptides, macrolides, sulfanilamides, ureidopenicillins. The anti-virals were remdesivir and acyclovir. The anti-mycotics were fluconazole and voriconazole.

| **Table S2:** | **Clinical Outcomes**  **per protocol analysis** |  |  |  |  |
| --- | --- | --- | --- | --- | --- |
| **Variate** | | | **Sevoflurane** | **Control** | **p-value** |
|  | | |  |  |  |
| **Primary outcome** | | | **n=28** | **n=26** |  |
| Primary endpoint reached, n (%) | | | 11(39) | 8(31) | 0.512 |
| Death, n (%) | | | 5(18) | 4(15) | 0.807 |
| Survivors | | | **n=23** | **n=22** |  |
| Persistent organ dysfunction (POD), n (%) | | | 6(26) | 4(18) | 0.522 |
| Need of mechanical ventilation | | | 6(26) | 4(18) | 0.522 |
| Need of vasopressors | | | 1(4) | 4(18) | 0.129 |
| Need of renal replacement therapy | | | 2(9) | 1(5) | 0.573 |

Data are presented as absolute numbers (n) and percentage (%). Abbreviations: POD: persistent organ dysfunction

**Table S3**

**Results of ventilatory parameters and ARDS co-interventions**

|  | Sevoflurane  **n=28** | Control  **n=32** | p-value |
| --- | --- | --- | --- |
| **Ventilatory parameters** |  |  |  |
| Minute ventilation (l/min) | 10.7±0.6 | 9.9±0.4 | **0.005** |
| PEEP (mbar) | 10.8 (10.2 to 11.5) | 10.4 (10.1 to 11.1) | 0.667 |
| FiO_2_ (%) | 46 (44 to 47) | 50 (47 to 54) | 0.050 |
| etCO_2_ (kPa) | 5.6±0.1 | 5.6±0.2 | 0.945 |
| Dynamic lung compliance (ml/mbar) | 50±10 | 53±6 | 0.403 |
|  |  |  |  |
| **Ventilator free days** (d) | 10.2 (0 to 20.6) | 11.6 (0 to 22.2) | 0.802 |
|  |  |  |  |
| **ARDS co-interventions** |  |  |  |
| Prone positioning n (%)  no  yes | 11(39)  17(61) | 11(34)  21(66) | 0.694 |
| ECMO support n (%)  no  yes | 27(96)  1(4) | 30(94)  2(6) | 0.635 |

Data are presented as mean±standard deviation, median and interquartile range, or absolute numbers and percentages. The ventilatory parameters and ARDS co-interventions have been evaluated over the first 8 days and include data from mechanically ventilated patients. Every day, the average values were recorded. Statistical differences have been calculated by Welch’s T-tests, Mann-Whitney, or by Chi-square tests. A p-value <0.05 is considered significant. Abbreviations: l: liters, min: minute, PEEP: peak expiratory pressure, %: percent, etCO2: end-tidal CO2, kPa: kilopascal, ml: milliliters, mbar: millibar, n: absolute numbers, ECMO: extracorporeal membrane oxygenation.

**Figure S1**

**Figure S1** illustrates the course of the oxygenation or the Horowitz Index (PaO_2_/FiO_2_) up to day 8 in patients subjected to continuous intravenous sedation or a 48h intervention with sevoflurane. The dots represent mean values; error bars indicate the standard deviation. Abbreviations: PaO_2_ = partial pressure of arterial oxygen saturation (in mmHg), FiO_2_ = fraction of inspired oxygen.

**Figure S2**

**Figure S2** shows the daily plasma values C-reactive protein (CRP, **S2A**) and procalcitonin (PCT, **S2B**). The dots represent mean values, error bars indicate the standard deviation.

**Figure S3**

**Figure S3** shows the daily mean norepinephrine dose required. The dots represent mean values; error bars indicate the standard deviation. The mean norepinephrine dose (overall) was 5.4±2.0 *vs.* 2.3±0.8 mcg/min in the sevoflurane *vs.* the control group (p=0.001).
